# 

**Published:** 1942

**Authors:** 


### CONTENTS.

*Page*

**The Thirty-first Long Fox Memorial Lecture: The Experimental Study of Development.** By Professor C. M. YONGE, D.Sc. .. .. .. .. .. .. .. .. 49

**Hospital Re-organization.** By SOMERVILLE HASTINGS, M.S., F.R.C.S. .. .. .. .. .. .. .. .. .. 61

**The Story of the Bristol Hospitals** .. .. .. .. .. 66

**Reviews of Books** .. .. .. .. .. .. .. .. .. 71

**Editorial Notes** .. .. .. .. .. .. .. .. .. 73

**Obituaries.** EDWARD FAWCETT, M.D. (Edin.), F.R.S. .. .. 77

CHARLES ARTHUR WIGAN, M.D. (Dur.), M.R.C.S., L.R.C.P. .. .. .. .. .. .. .. 78

ELIAS GEORGE HALL, M.B. (Lond.), M.B., Ch.B. (Brist.), M.R.C.S., L.R.C.P. .. .. .. .. .. 79

**The Medical Library of the University of Bristol** .. .. .. 79

**Publications Received** .. .. .. .. .. .. .. 82

**Local Medical Notes** .. .. .. .. .. .. .. 82

